# Supplementary material for: Inequalities in healthcare use during the COVID-19 pandemic
Source: Nat Commun. 2024 Feb 29;15:1894. doi: 10.1038/s41467-024-45720-2 (PMC10904793; doi:10.1038/s41467-024-45720-2)
Supplement: Supplementary file 1 — Supplementary Information [file 41467_2024_45720_MOESM1_ESM.pdf]

Supplementary Information

		Population		Covid-19 deaths (prop)	
		2020	2021	2020	2021
Total (N)		14,071,420	14,164,191	20,160	19,590
Gender (%)	Men	0.49	0.49	10,770 (0.53)	10,780 (0.55)
	Women	0.51	0.51	9,390 (0.47)	8,810 (0.45)
Age Group (%)	18-29	0.19	0.19	10 (0.0)	15 (0.0)
	30-65	0.58	0.58	1,280 (0.06)	1,705 (0.09)
	66-75	0.13	0.14	3,345 (0.17)	3.790 (0.19)
	76+	0.09	0.09	15,530 (0.77)	14,080 (0.72)
Migrant background (%)	No migrant background	0.76	0.76	16,820 (0.83)	16,005 (0.82)
	Migrant background	0.24	0.24	3,340 (0.17)	3,585 (0.18)
Poverty (%)	Above poverty line	0.91	0.91	17,025 (0.84)	15,975 (0.82)
	Below poverty line	0.09	0.09	3,135 (0.16)	3,620 (0.19)

Table SI-1: COVID-19 related mortality by sociodemographic characteristics in 2020. *Note*: counts by age group have been rounded to multiples of five given small sample sizes. As a result, the total count across some demographic variables is higher than the overall total reported in the first row.

		2017	2018	2019	2020	2021
Total (N)		13,677,385	13,796,082	13,925,588	14,071,420	14,164,191
Gender (%)	Women	0.51	0.51	0.51	0.51	0.51
	Men	0.49	0.49	0.49	0.49	0.49
Age Group (%)	18-29	0.19	0.19	0.19	0.19	0.19
	30-65	0.60	0.59	0.59	0.58	0.58
	66-75	0.13	0.13	0.13	0.13	0.14
	76+	0.09	0.09	0.09	0.09	0.09
Migrant background (%)	No migrant background	0.78	0.78	0.77	0.76	0.76
	Migrant background	0.22	0.22	0.23	0.24	0.24
Poverty (%)	Above poverty line	0.91	0.91	0.91	0.91	0.91
	Below poverty line	0.09	0.09	0.09	0.09	0.09

Table SI-2: Adult population composition The Netherlands between 2017 and 2020.

Variable	Description
Age	
18 to 29	Individual is aged between 18 and 29 (inclusive) at the start of the year
30 to 65	Individual is aged between 30 and 65 (inclusive) at the start of the year
66 to 75	Individual is aged between 66 and 75 (inclusive) at the start of the year
76 or older	Individual is aged 76 or older at the start of the year
Migrant background	
No migrant background	Individual and their mother was born in The Netherlands
Migrant background	Individual and / or their mother was born outside of The Netherlands
Sex	
Female	Individual's registered sex at birth was female
Male	Individual's registered sex at birth was male
Poverty*	
Above poverty line	Individual's household income is 120% of the social minimum or less
Below poverty line	Individual's household income is above 120% of the social minimum

* The cutoff point of 120% of the social minimum is used to determine eligibility for benefits in The Netherlads. The social minimum further depends on the household composition, more accurately reflecting the needs of a household than an absolute cutoff.

Table SI-3: Sociodemographic variables included in the data.

	2017	2018	2019	2020	2021
Total	30,148,104	32,912,952	33,530,296	30,802,000	33,127,418
High urgency	6,155,880	6,426,136	6,468,586	5,937,053	6,228,617
Middle urgency	7,730,247	8,773,125	8,878,915	8,376,163	9,039,804
Low urgency	13,915,943	15,157,978	15,425,392	13,843,809	14,947,447
No urgency	4,291,290	4,839,605	5,082,821	4,768,462	5,209,291

Table SI-4: Total count of individuals treated per week between 2017 and 2020 by urgency type. *Note*: An individual is counted as having received treatment in a given week at a given urgency level if they underwent at least one health activity with that urgency level that week. If an individual received both a high urgency and a low urgency treatment within a given week, they will appear in both urgency counts.

		2017	2018	2019	2020	2021
Total (N)		88,790,300	99,581,536	100,792,648	89,334,611	95,464,293
Gender (%)	Women	0.55	0.54	0.54	0.54	0.54
	Men	0.45	0.46	0.46	0.46	0.46
Age Group (%)	18-29	0.07	0.07	0.07	0.07	0.07
	30-65	0.48	0.47	0.46	0.46	0.46
	66-75	0.23	0.24	0.24	0.24	0.24
	76+	0.22	0.22	0.23	0.23	0.22
Migrant background (%)	Non-migrant background	0.82	0.81	0.81	0.81	0.80
	Migrant background	0.18	0.19	0.19	0.19	0.20
Poverty (%)	Above poverty line	0.86	0.86	0.87	0.87	0.87
	Below poverty line	0.14	0.14	0.13	0.13	0.31

Table SI-5: Total count of treatment activities between 2017 and 2020 and by demographic group

	2017	2018	2019	2020	2021

Total	88,790,300	99,581,536	100,792,648	89,334,611	95,464,293
High urgency	30,617,208	33,694,326	33,877,892	30,201,885	31,282,407
Middle urgency	17,442,952	20,473,180	20,604,680	18,894,814	20,496,416
Low urgency	28,449,024	31,442,997	31,708,846	26,968,825	29,276,954
No urgency	12,281,116	13,971,033	14,601,230	13,269,087	14,408,516

Table SI-6: Total count of treatment activities between 2017 and 2020 and by urgency type

		2017	2018	2019	2020	2021
Average patients per week (N)		579,771	632,941	644,813	592,346	637,066
Gender (%)	Women	0.56	0.56	0.56	0.55	0.56
	Men	0.44	0.44	0.44	0.45	0.44
Age Group (%)	18-29	0.08	0.08	0.08	0.08	0.08
	30-65	0.52	0.51	0.51	0.50	0.50
	66-75	0.23	0.23	0.23	0.23	0.23
	76+	0.17	0.18	0.18	0.18	0.18
Migrant background (%)	No migrant background	0.81	0.80	0.80	0.80	0.80
	Migrant background	0.19	0.20	0.20	0.20	0.20
Poverty (%)	Above poverty line	0.88	0.88	0.88	0.89	0.89
	Below poverty line	0.12	0.12	0.12	0.11	0.11

Table SI-7: Average number of individuals treated per week between 2017 and 2020 (based on 52 weeks) and by demographic group. *Note*: An individual is counted as having received treatment in a given week if they underwent at least one health activity that week. If an individual received at least one health activity per week across two weeks, they will be counted twice.

Sociodemographic group	2020 (prop)	2021 (prop)
18 to 29, Female, No migrant background, Above poverty line	867,258	868,716
18 to 29, Female, No migrant background, Below poverty line	36,922	35,226
18 to 29, Female, Migrant background, Above poverty line	369,381	369,656
18 to 29, Female, Migrant background, Below poverty line	40,515	39,590
18 to 29, Male, No migrant background, Above poverty line	915,422	915,969
18 to 29, Male, No migrant background, Below poverty line	30,592	29,538
18 to 29, Male, Migrant background, Above poverty line	373,343	377,070
18 to 29, Male, Migrant background, Below poverty line	38,346	36,905
30 to 65, Female, No migrant background, Above poverty line	2,853,351	2,843,724
30 to 65, Female, No migrant background, Below poverty line	195,255	188,059
30 to 65, Female, Migrant background, Above poverty line	874,617	905,639
30 to 65, Female, Migrant background, Below poverty line	178,598	177,410
30 to 65, Male, No migrant background, Above poverty line	2,960,374	2,949,226
30 to 65, Male, No migrant background, Below poverty line	146,691	141,424
30 to 65, Male, Migrant background, Above poverty line	862,432	897,417
30 to 65, Male, Migrant background, Below poverty line	142,595	140,688
66 to 75, Female, No migrant background, Above poverty line	737,249	746,655
66 to 75, Female, No migrant background, Below poverty line	78,884	78,100
66 to 75, Female, Migrant background, Above poverty line	102,120	106,003

66 to 75, Female, Migrant background, Below poverty line	38,990	41,046
66 to 75, Male, No migrant background, Above poverty line	742,553	752,099
66 to 75, Male, No migrant background, Below poverty line	48,339	48,810
66 to 75, Male, Migrant background, Above poverty line	101,513	103,842
66 to 75, Male, Migrant background, Below poverty line	28,129	29,255
76+, Female, No migrant background, Above poverty line	539,150	551,528
76+, Female, No migrant background, Below poverty line	123,507	124,170
76+, Female, Migrant background, Above poverty line	68,951	70,009
76+, Female, Migrant background, Below poverty line	27,664	28,801
76+, Male, No migrant background, Above poverty line	425,967	442,115
76+, Male, No migrant background, Below poverty line	47,945	48,506
76+, Male, Migrant background, Above poverty line	56,248	57,672
76+, Male, Migrant background, Below poverty line	18,519	19,323

Table SI-8: Counts of fully interacted demographic groups at the start of 2020 and 2021.

**Variable**	**Count**
Age	
18 to 29	2,080
30 to 65	2,080
66 to 75	2,080
76+	2,080
Sex	

Female	4,160
Male	4,160
Poverty	
Above poverty line	4,160
Below poverty line	4,160
Migrant background	
No migrant background	4,160
Migrant background	4,160
Weekly holidays	
0	7,456
1	448
2	416
Pandemic indicators	
Pandemic	2,976
Pandemic: Wave 1	352
Pandemic: Wave 2	1,216
Pandemic: Wave 3	352
Pandemic: Interwave 1	544
Pandemic: Interwave 2	512
Total	8,320

Table SI-9: Descriptive statistics of the dataset used for multivariate analysis, containing 32 timeseries at the weekly level for the period 2017-2021. Week and year variables are omitted from the table for brevity.

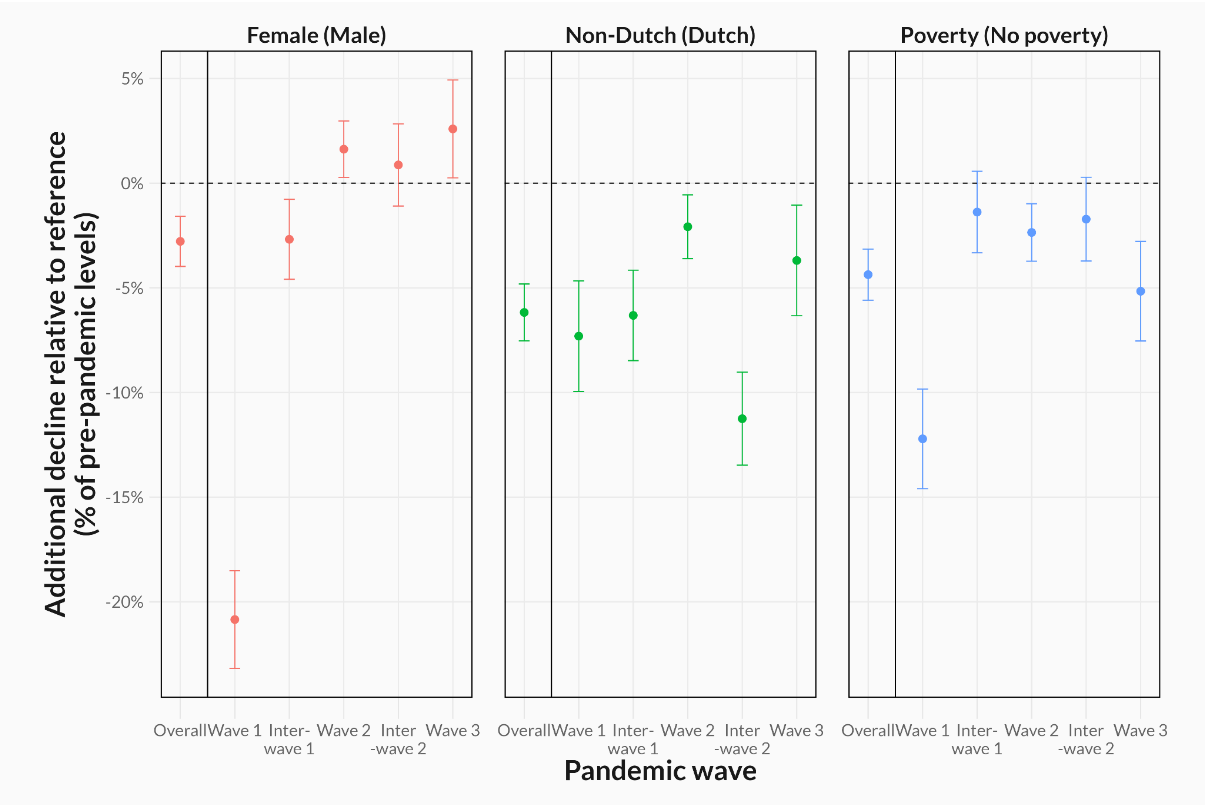

Figure SI-1: Regression coefficients showing additional declines in weekly non-COVID patients for demographic subgroups relative to a reference group. Each coefficient is scaled by that group's pre-pandemic weekly average. Linear regression is performed on 32 weekly timeseries for the period 2017-2021 reflecting each unique fully interacted demographic group. We include week, year and holiday controls, demographic covariates and random effects at the group level. Depicted coefficients show the interactions between each demographic variable and a single pandemic dummy ('Overall') as well as dummies for each individual COVID wave (see Methods). Error bars around estimates indicate standard errors.

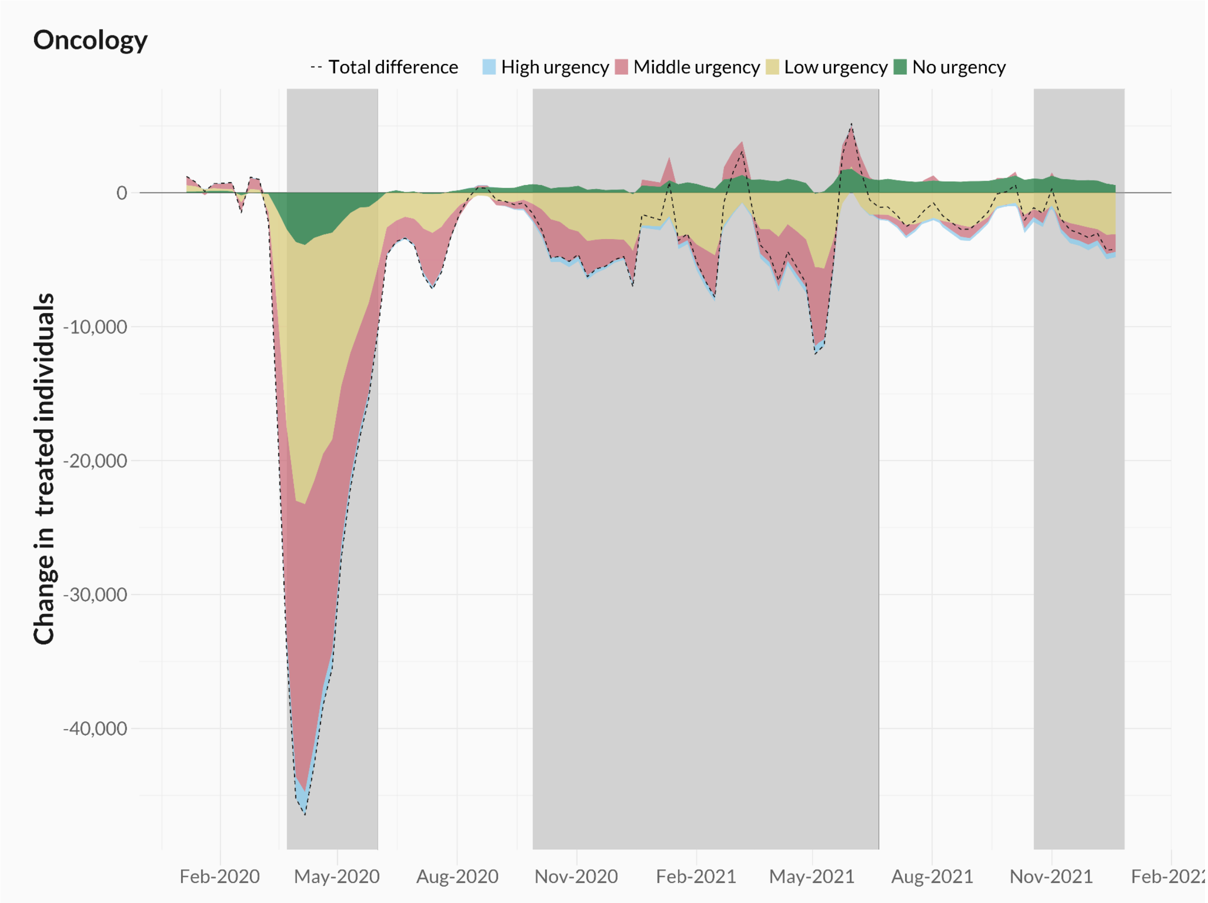

Figure SI-2a: Difference between the observed and predicted number of treated individuals per week in 2020, for oncological care. Colours differentiate between urgency types (high, middle, low, and no urgency). COVID hospital waves are depicted in shaded grey.

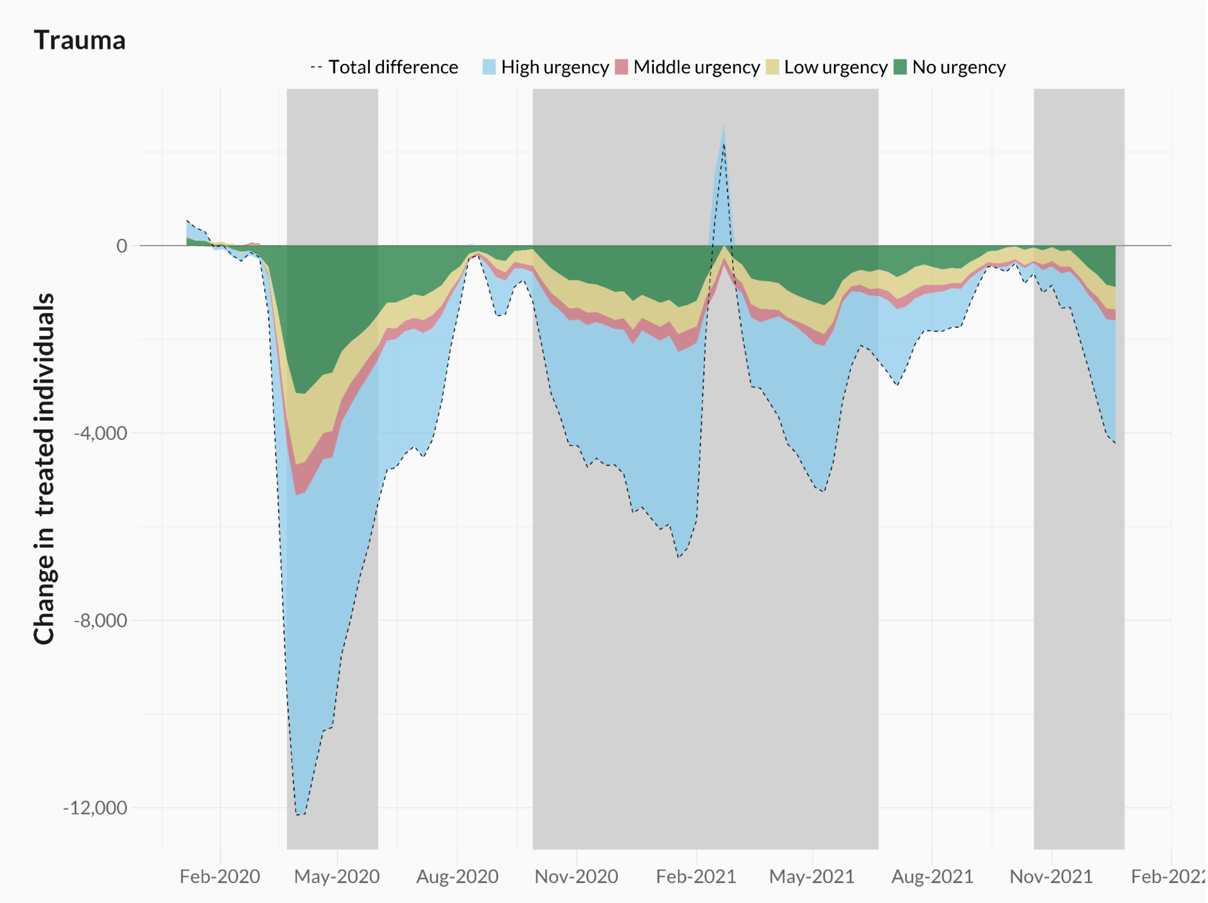

Figure SI-2b: Difference between the observed and predicted number of treated individuals per week in 2020, for trauma care. Colours differentiate between urgency types (high, middle, low, and no urgency). COVID hospital waves are depicted in shaded grey.

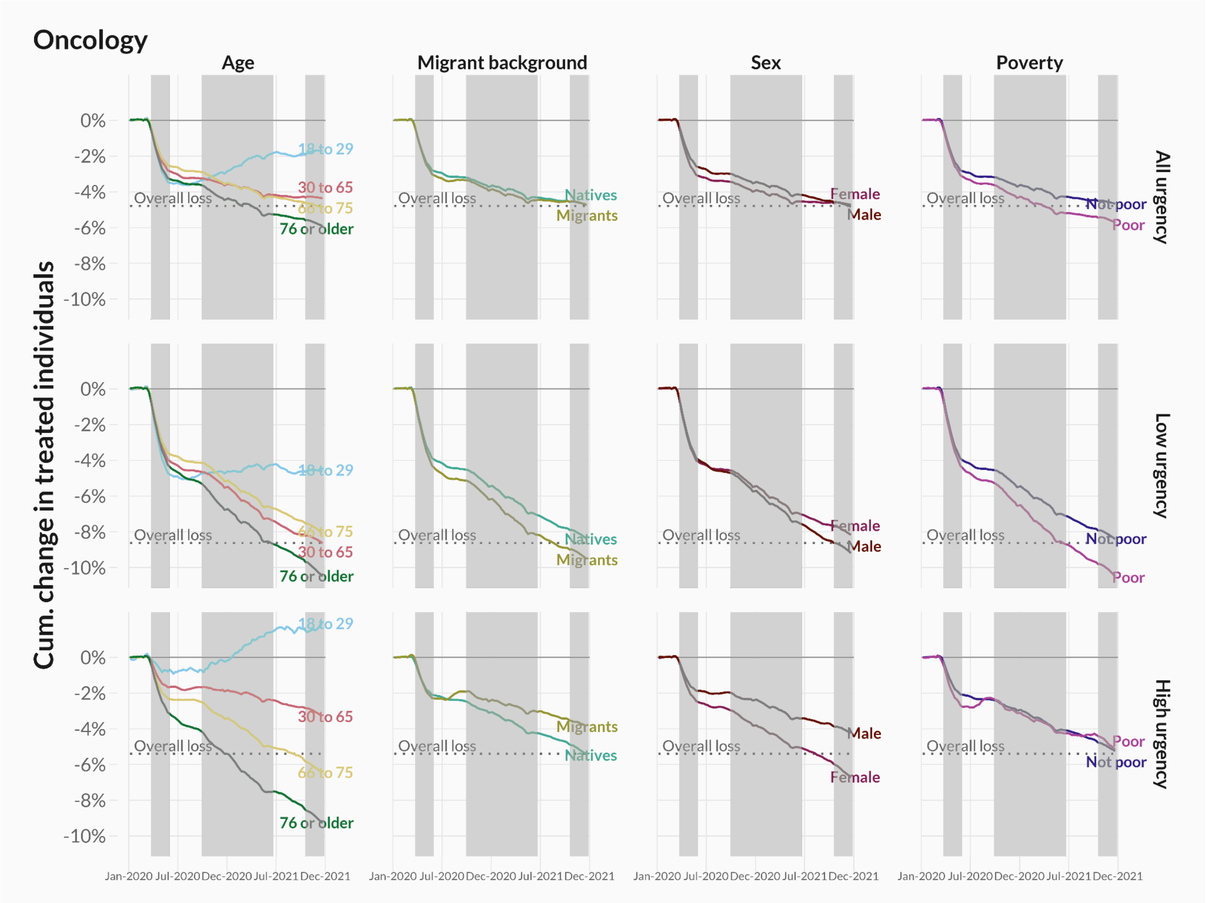

Figure SI-3a: Cumulative age- and sex-adjusted difference between the observed and predicted number of treated individuals in 2020 and 2020, across urgency types (rows) for all demographic groups (columns), for treatments related to oncology care. COVID hospital wavesCovid hospital waves are depicted in shaded grey.

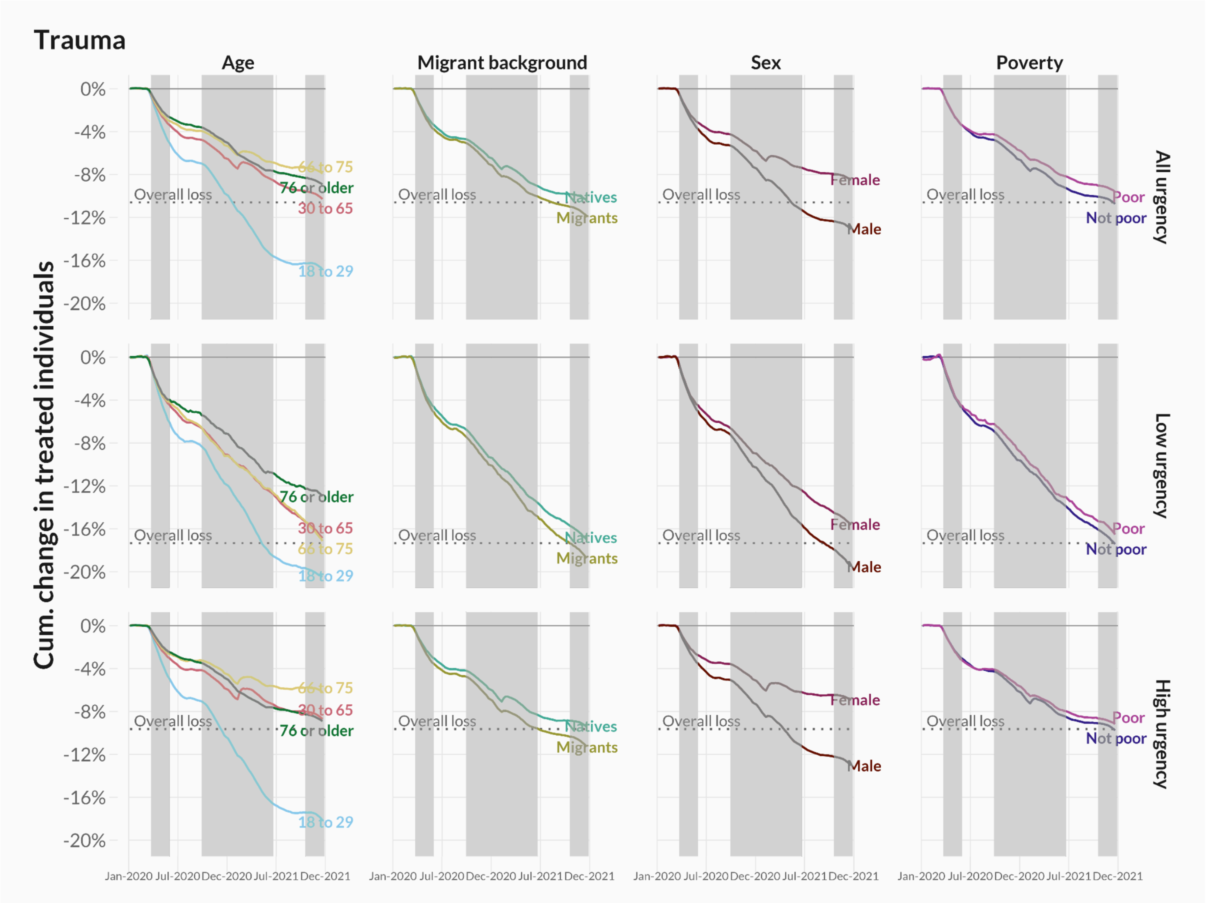

Figure SI-3b: Cumulative age- and sex-adjusted difference between the observed and predicted number of treated individuals in 2020 and 2020, across urgency types (rows) for all demographic groups (columns), for treatments related to trauma care. COVID hospital wavesCovid hospital waves are depicted in shaded grey.

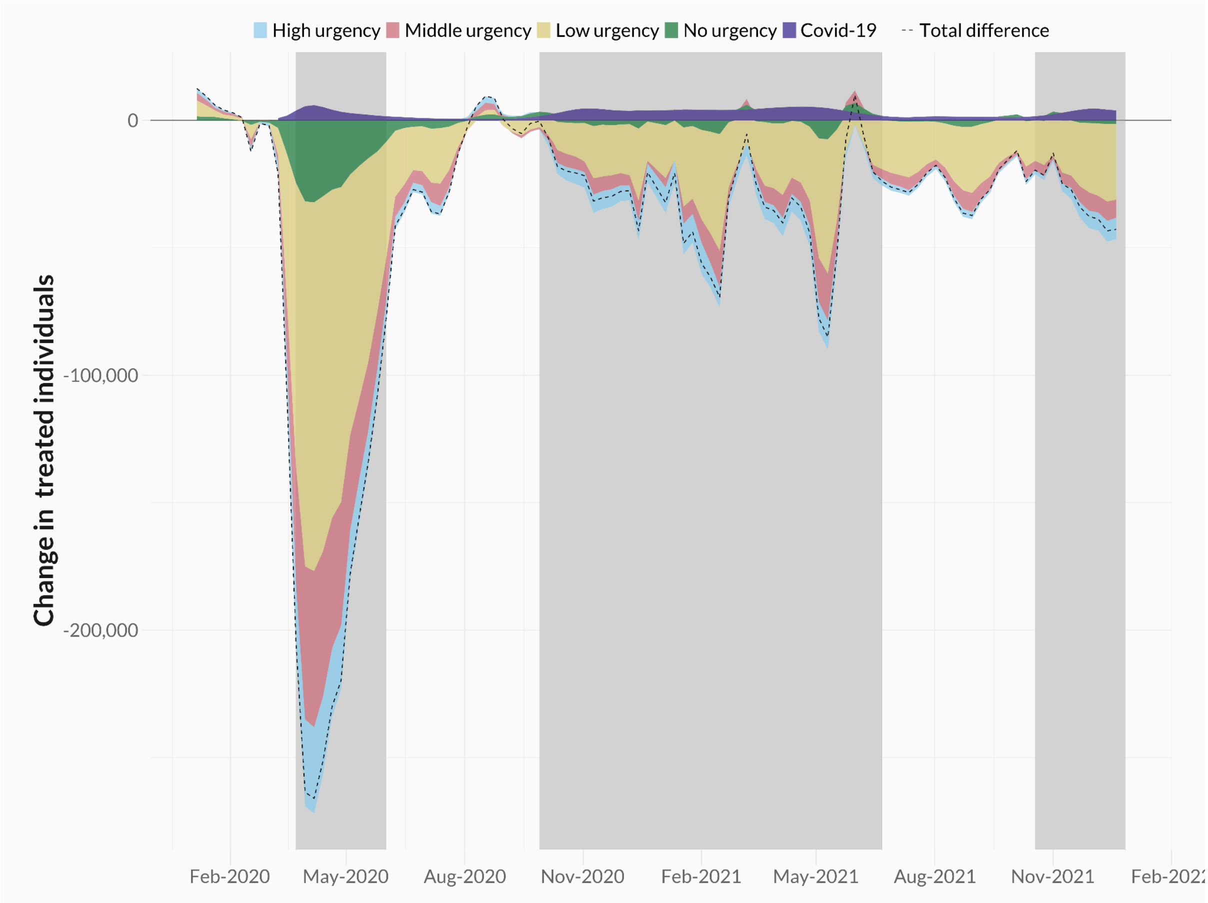

Figure SI-4a: Difference between the observed and predicted number of treated individuals per week in 2020 and 2021, when only including individuals receiving healthcare procedures involving outpatient visits, clinical activities, ER activities and diagnostics but excluding activities related to laboratory medicine. Colours differentiate between urgency types (high, middle, low, and no urgency). COVID hospital waves are depicted in shaded grey.

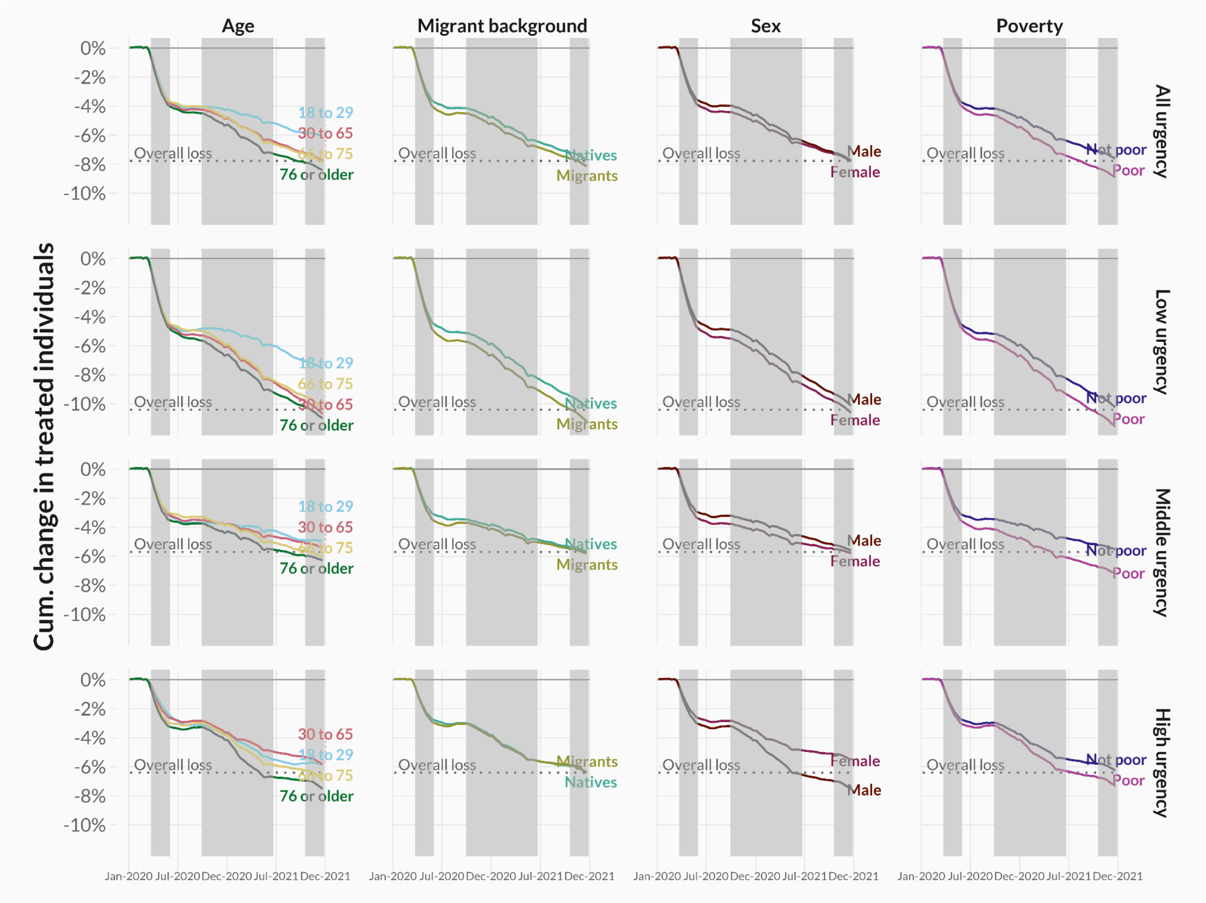

Figure SI-4b: Cumulative age- and sex-adjusted difference between the observed and predicted number of treated individuals in 2020 and 2021 when only including individuals receiving healthcare procedures involving outpatient visits, clinical activities, ER activities and diagnostics but excluding activities related to laboratory medicine, across urgency types (rows) and demographic groups (columns). COVID hospital waves are depicted in shaded grey.

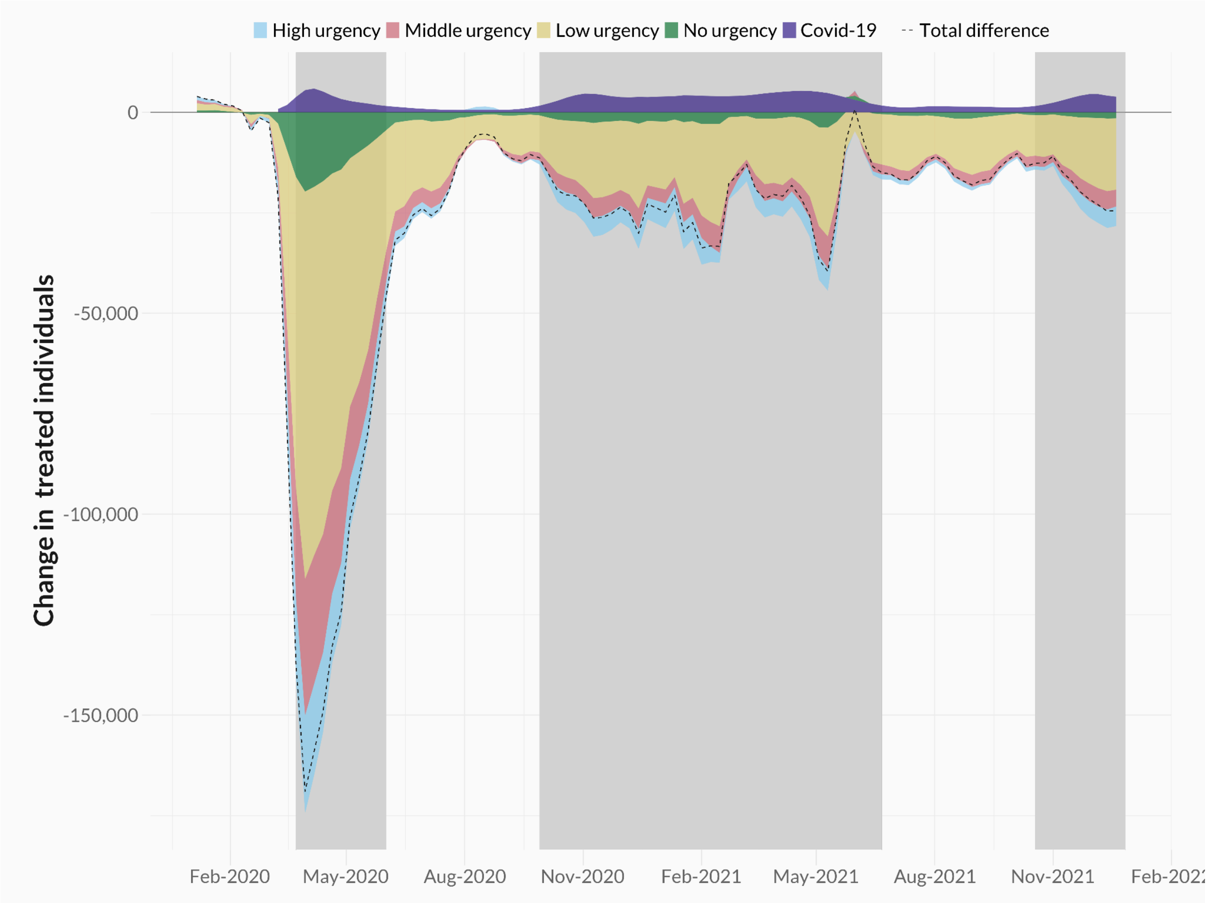

Figure SI-5a: Difference between the observed and predicted number of treated individuals per week in 2020 and 2021, when only including individuals receiving healthcare procedures involving clinical and / or ER activities. Colours differentiate between urgency types (high, middle, low, and no urgency). COVID hospital wavesCovid hospital waves are depicted in shaded grey. Values depict three week moving averages.

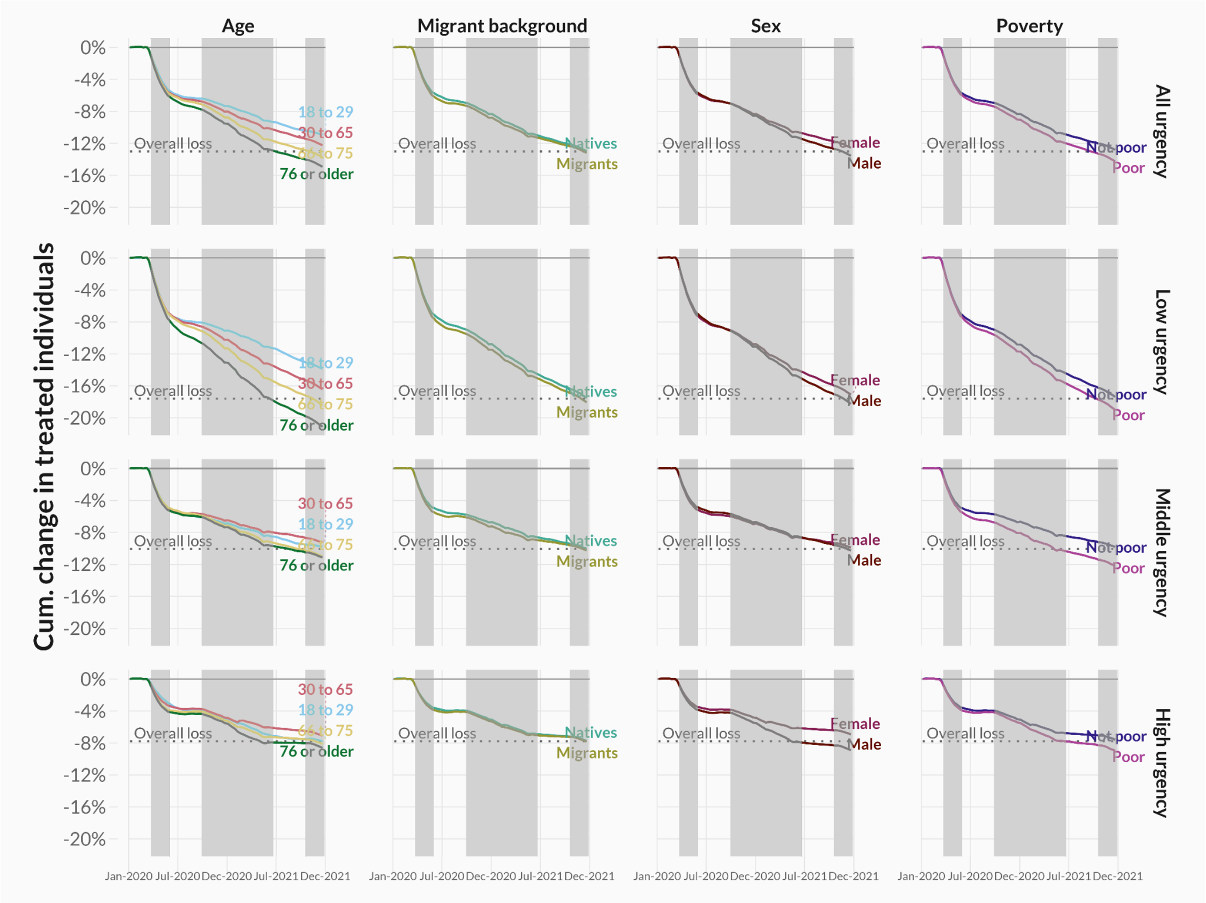

Figure SI-5b: Cumulative age- and sex-adjusted difference between the observed and predicted number of treated individuals in 2020 and 2021 when only including individuals receiving healthcare procedures involving clinical and / or ER activities, across urgency types (rows) and demographic groups (columns). Covid hospital waves are depicted in shaded grey.

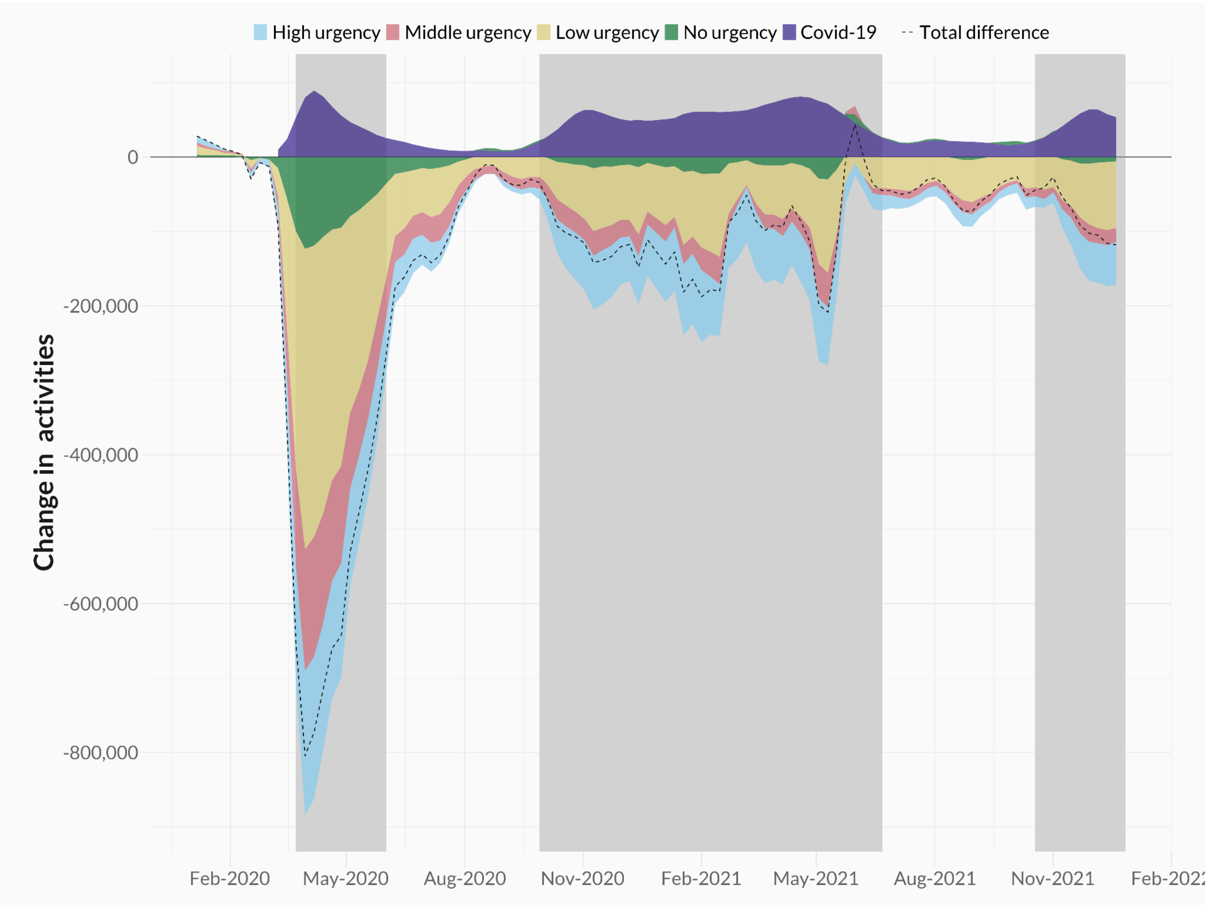

Figure SI-6a: Difference between the observed and predicted number of healthcare activities per week in 2020. Colours differentiate between urgency types (high, middle, low, and no urgency). COVID hospital waves are depicted in shaded grey. Values depict three week moving averages.

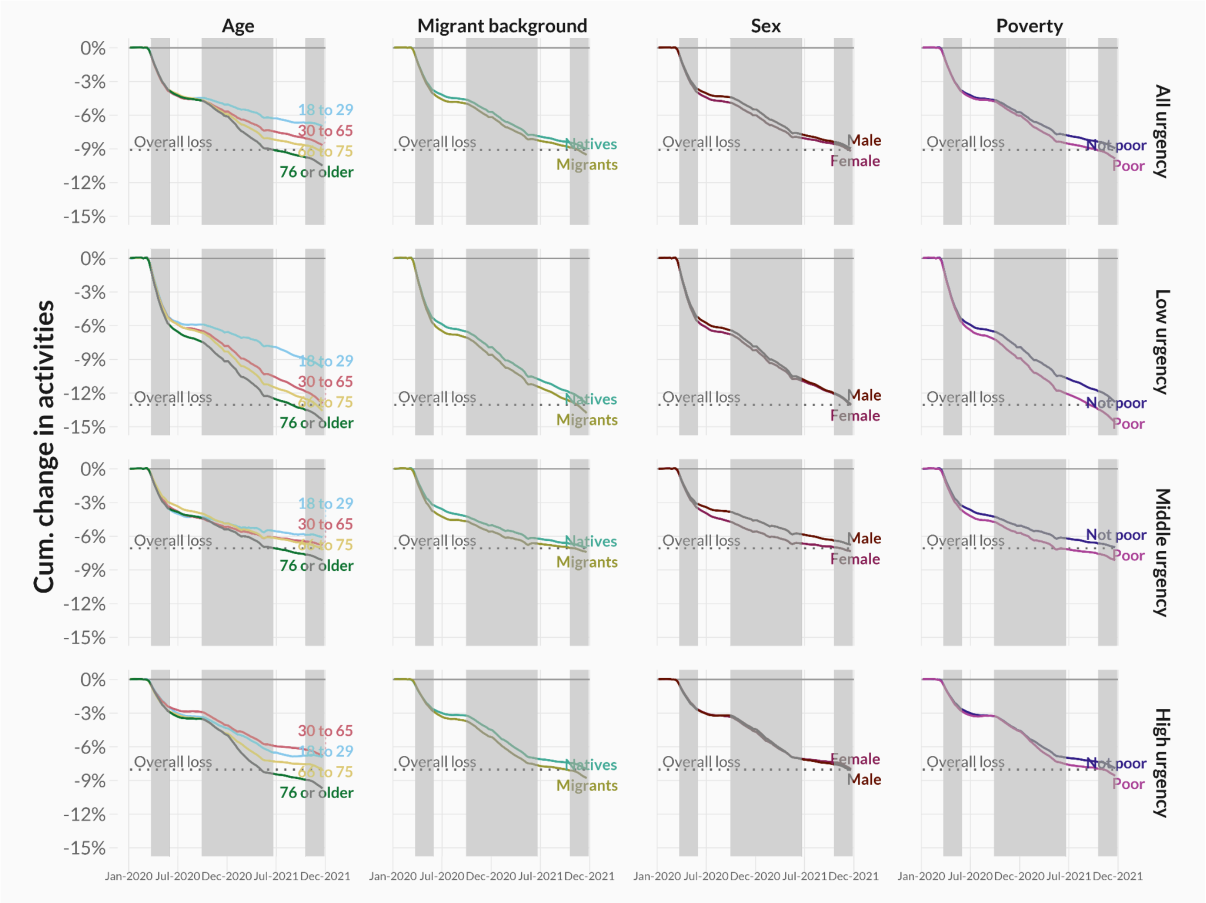

Figure SI-6b: Cumulative age- and sex-adjusted difference between the observed and predicted number of activities in 2020 and 2021, across urgency types (rows) and demographic groups (columns). COVID hospital waves are depicted in shaded grey.

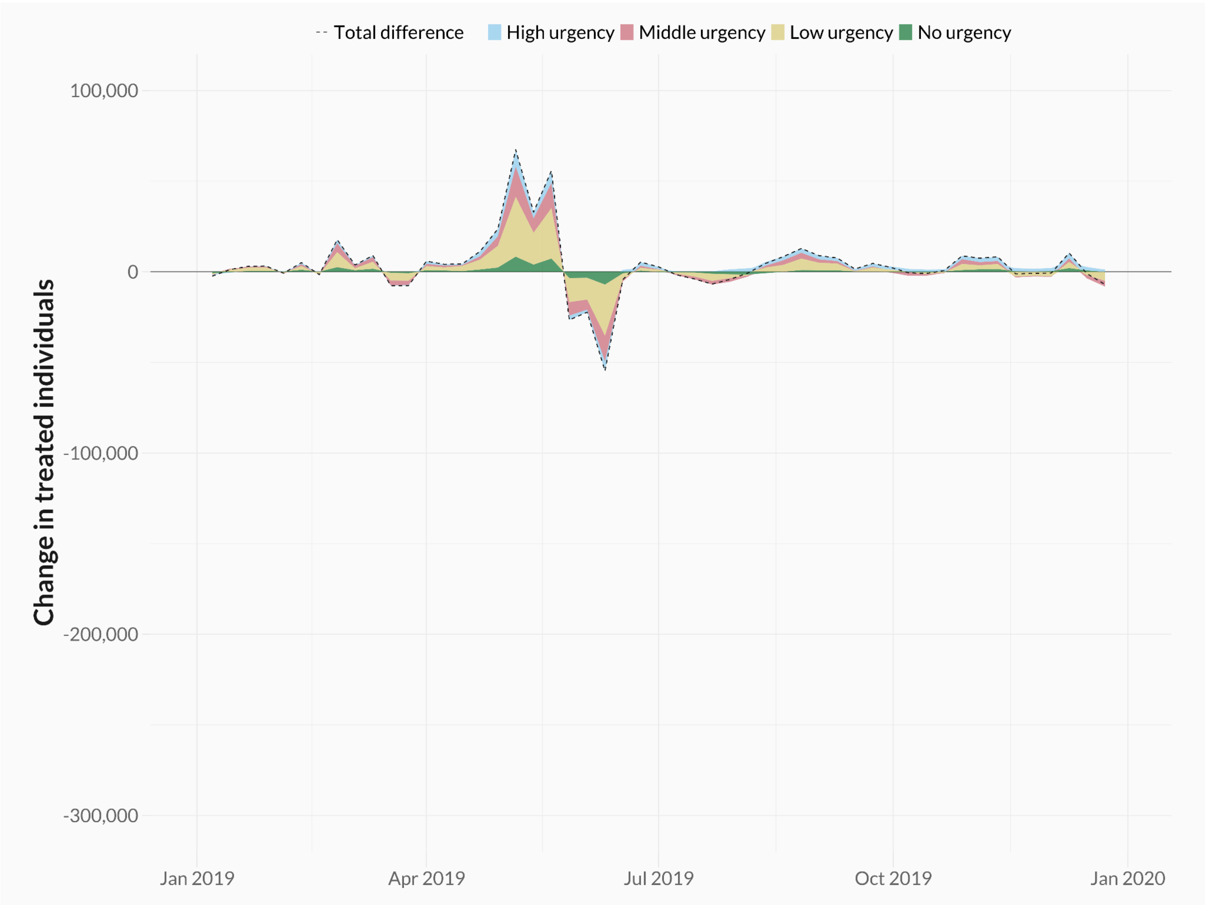

Figure SI-7a: Difference between the observed and predicted number of treated individuals per week in 2019. Colours differentiate between urgency types (high, middle, low, and no urgency). Values depict three week moving averages.

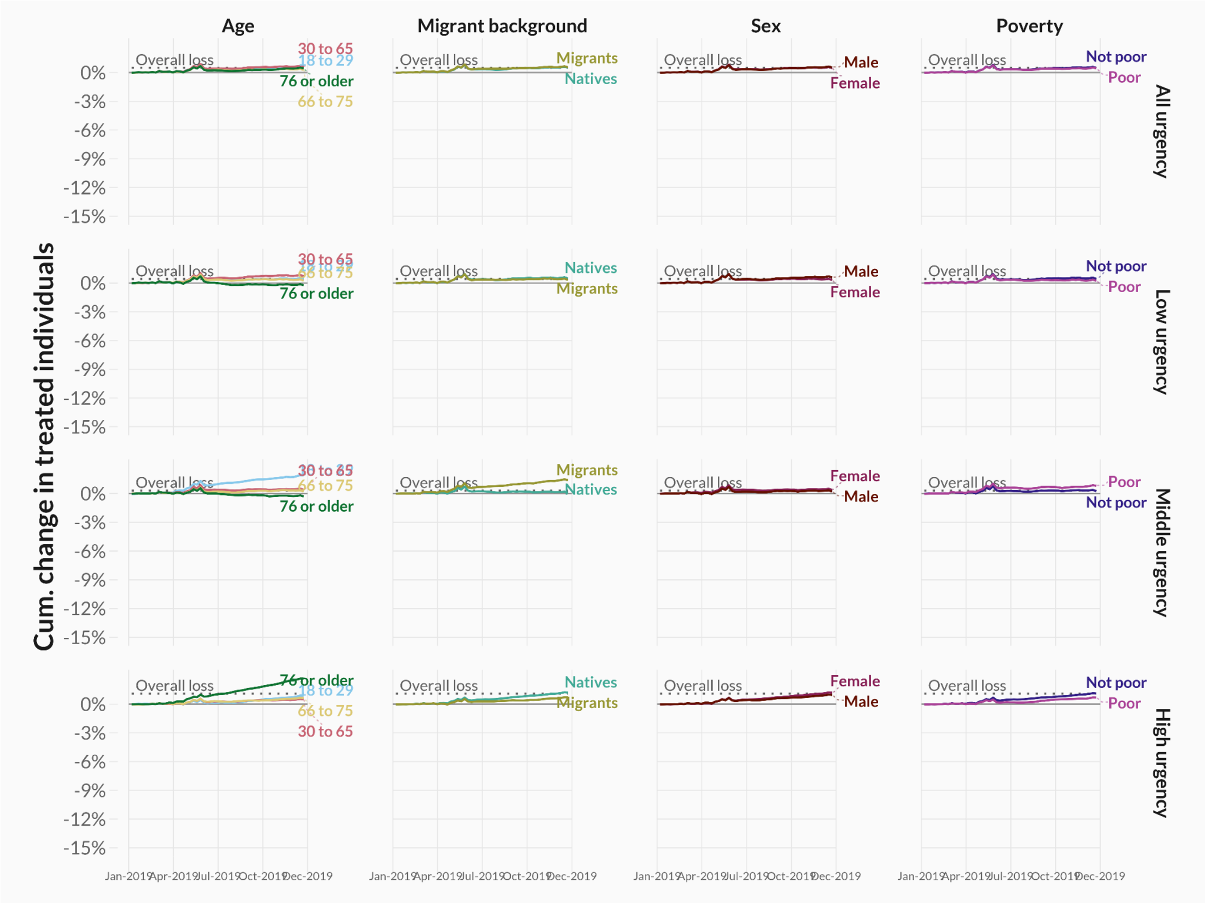

Figure SI-7b: Cumulative age- and sex-adjusted difference in observed versus expected healthcare users by urgency (rows) and demographic groups (columns) in 2019.

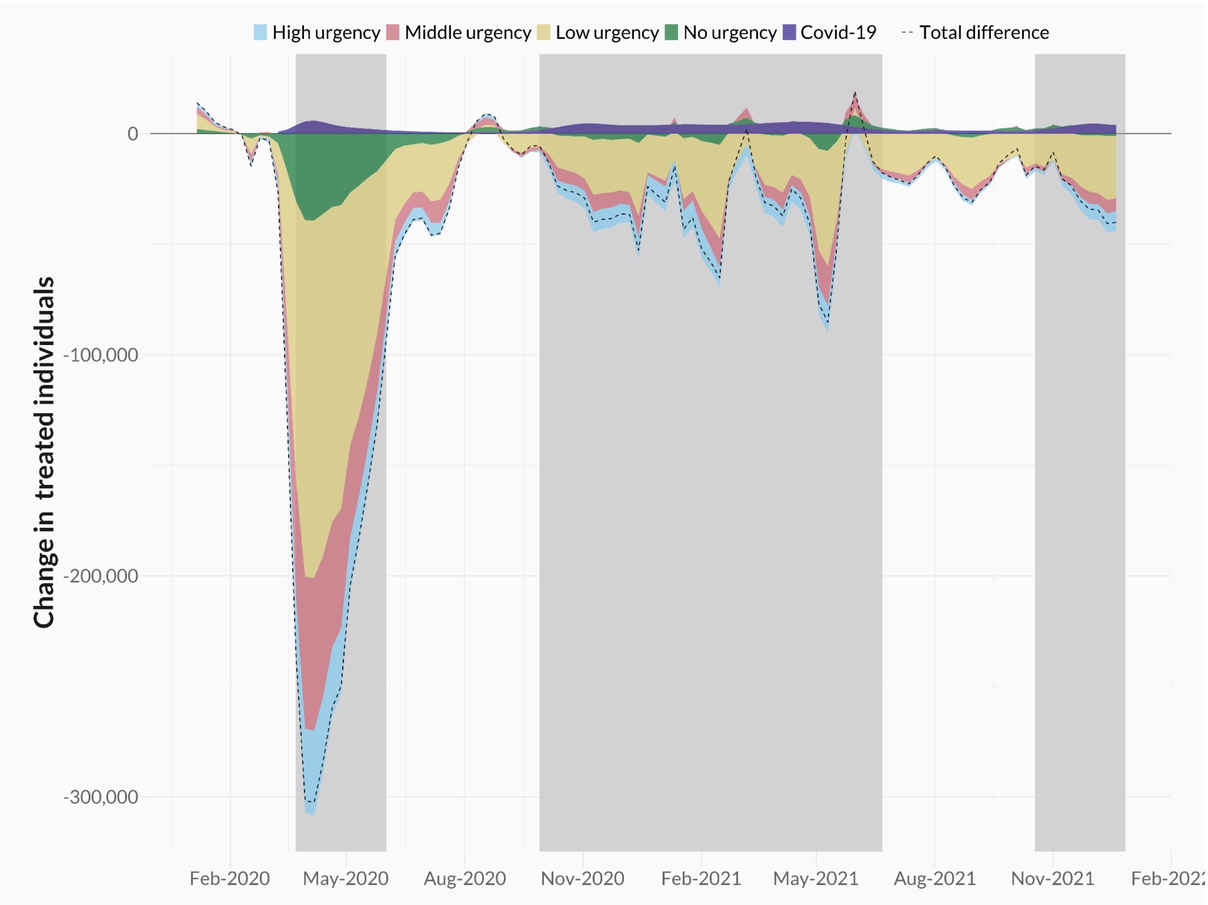

Figure SI-8a: Difference between the observed and predicted number of treated individuals per week in 2020 and 2021 when using a negative binomial regression to make weekly predictions. Colours differentiate between urgency types (high, middle, low, and no urgency) and patients treated for COVID-19. COVID hospital waves are depicted in shaded grey.

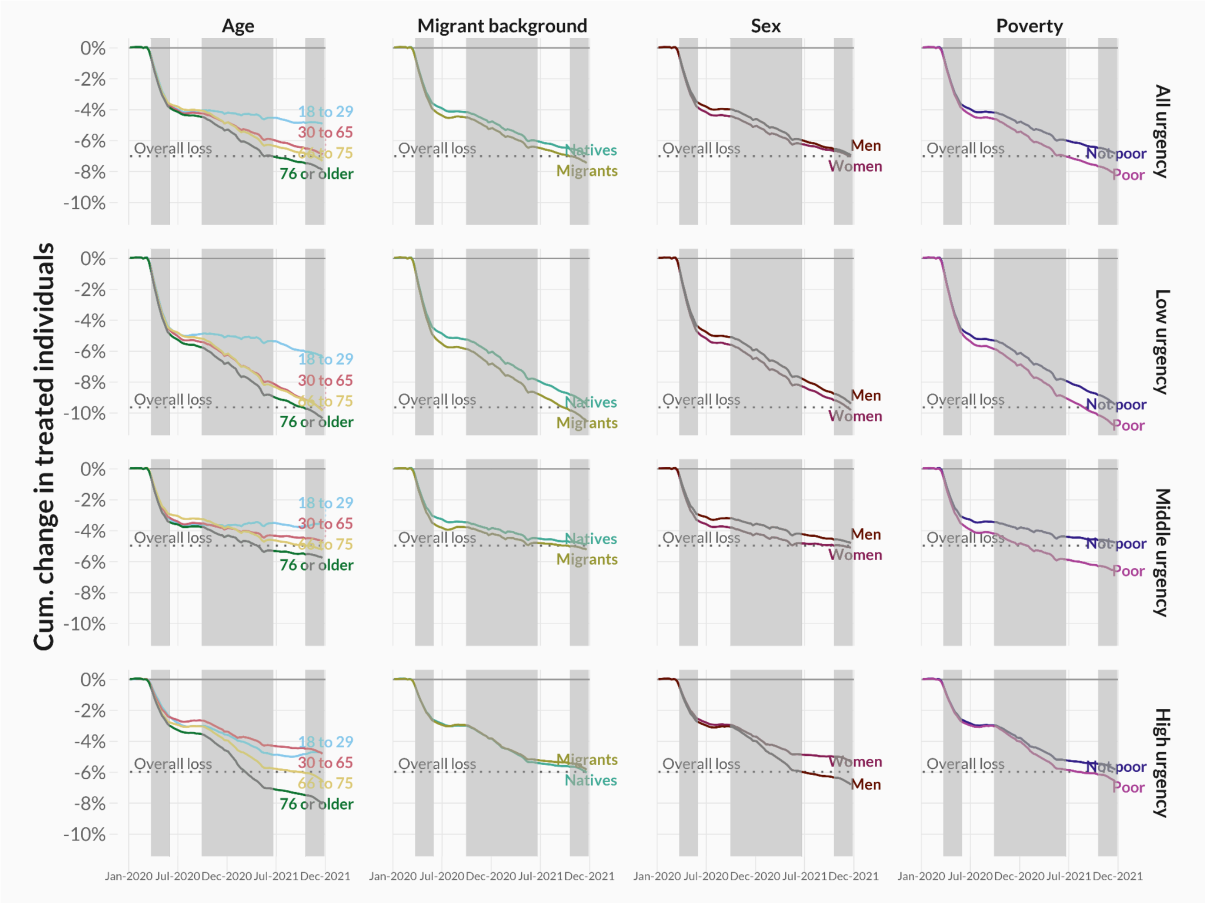

Figure SI-8b: Cumulative age- and sex-adjusted differences between the observed and predicted number of treated individuals in 2020 and 2021, across urgency types (rows) and demographic groups (columns) when using a negative binomial regression to make weekly predictions. COVID hospital waves are depicted in shaded grey.
