# Supplementary material for: Inequalities in healthcare use during the COVID-19 pandemic
Source: Nat Commun. 2024 Feb 29;15:1894. doi: 10.1038/s41467-024-45720-2 (PMC10904793; doi:10.1038/s41467-024-45720-2)
Supplement: Supplementary file 3 — Reporting Summary [file 41467_2024_45720_MOESM3_ESM.pdf]

nature portfolio

Corresponding author(s): Mark D. Verhagen

Last updated by author(s): 01/26/2024

Reporting Summary

Nature Portfolio wishes to improve the reproducibility of the work that we publish. This form provides structure for consistency and transparency in reporting. For further information on Nature Portfolio policies, see our Editorial Policies and the Editorial Policy Checklist.

Statistics

For all statistical analyses, confirm that the following items are present in the figure legend, table legend, main text, or Methods section.

n/a	Confirmed	
☐	☒	The exact sample size (*n*) for each experimental group/condition, given as a discrete number and unit of measurement
☐	☒	A statement on whether measurements were taken from distinct samples or whether the same sample was measured repeatedly
☒	☐	The statistical test(s) used AND whether they are one- or two-sided<br>*Only common tests should be described solely by name; describe more complex techniques in the Methods section.*
☐	☒	A description of all covariates tested
☐	☒	A description of any assumptions or corrections, such as tests of normality and adjustment for multiple comparisons
☐	☒	A full description of the statistical parameters including central tendency (e.g. means) or other basic estimates (e.g. regression coefficient) AND variation (e.g. standard deviation) or associated estimates of uncertainty (e.g. confidence intervals)
☒	☐	For null hypothesis testing, the test statistic (e.g. *F*, *t*, *r*) with confidence intervals, effect sizes, degrees of freedom and *P* value noted<br>*Give P values as exact values whenever suitable.*
☒	☐	For Bayesian analysis, information on the choice of priors and Markov chain Monte Carlo settings
☒	☐	For hierarchical and complex designs, identification of the appropriate level for tests and full reporting of outcomes
☒	☐	Estimates of effect sizes (e.g. Cohen's *d*, Pearson's *r*), indicating how they were calculated

Our web collection on statistics for biologists contains articles on many of the points above.

Software and code

Policy information about availability of computer code

Data collection	Instruction for data collection can be found on our GitHub page: https://github.com/MarkDVerhagen/Dutch_healthcare_inequalities_COVID19
Data analysis	Code used to analyse the data can be found on our GitHub page: https://github.com/MarkDVerhagen/Dutch_healthcare_inequalities_COVID19

For manuscripts utilizing custom algorithms or software that are central to the research but not yet described in published literature, software must be made available to editors and reviewers. We strongly encourage code deposition in a community repository (e.g. GitHub). See the Nature Portfolio guidelines for submitting code & software for further information.

Data

Policy information about availability of data

All manuscripts must include a data availability statement. This statement should provide the following information, where applicable:

- Accession codes, unique identifiers, or web links for publicly available datasets
- A description of any restrictions on data availability
- For clinical datasets or third party data, please ensure that the statement adheres to our policy

All results presented here are calculated from non-public registry data from Centraal Bureau voor de Statistiek (CBS), accessed through the Remote Access environment. CBS was not involved in the calculation of any of the results presented here. While the data are not publicly available, academic institutions can apply

for access through the CBS.

Research involving human participants, their data, or biological material

Policy information about studies with human participants or human data. See also policy information about sex, gender (identity/presentation), and sexual orientation and race, ethnicity and racism.

Reporting on sex and gender	We included Sex as a demographic variable in our analysis, which is defined as 'registered sex' by administrative records.
Reporting on race, ethnicity, or other socially relevant groupings	We included non-native Dutch and native Dutchindividuals as demographic variables, which are defined relative to the birth country of the individual and their mother (when either is not in The Netherlands, the individual is classified as 'non-native Dutch')
Population characteristics	The covariate-relevant population characteristics of the data consists of: an indicator whether an individual's household income was classified to be below or above the poverty line, their sex at birth, their age, whether they or their mother was born in a country other than The Netherlands.
Recruitment	We use population registry data, thus no active recruitment was undertaken
Ethics oversight	Research Ethics Committee of Department of Sociology at the University of Oxford

Note that full information on the approval of the study protocol must also be provided in the manuscript.

Field-specific reporting

Please select the one below that is the best fit for your research. If you are not sure, read the appropriate sections before making your selection.

☐ Life sciences ☒ Behavioural & social sciences ☐ Ecological, evolutionary & environmental sciences

For a reference copy of the document with all sections, see nature.com/documents/nr-reporting-summary-flat.pdf

Behavioural & social sciences study design

All studies must disclose on these points even when the disclosure is negative.

Study description	Quantitative analysis to estimate declines in non-COVID healthcare use of the entire population in The Netherlands.
Research sample	The entire adult population of The Netherlands in the period 2017-2021 was used. Since the target population of interest is the entire adult population of The Netherlands, the sample is fully representative of the target population. The dataset was provided by Statistics Netherlands (CBS) and is available to researchers that are granted access to the CBS' Remote Access environment.
Sampling strategy	The full population of people registered in The Netherlands was used as a sample, in other words there was complete sampling of the target population of interest.
Data collection	Via administrative registry (CBS), participants were blinded to experimental conditions and the study hypothesis.
Timing	2017 through 2021.
Data exclusions	No exclusion criteria were implemented relative to the target population and sample thereof.
Non-participation	No participants were involved in the study.
Randomization	Since we implemented a complete sampling of the population of interest, and the participants were not exposed to any experimental condition, randomization is not applicable to the study.

Reporting for specific materials, systems and methods

We require information from authors about some types of materials, experimental systems and methods used in many studies. Here, indicate whether each material, system or method listed is relevant to your study. If you are not sure if a list item applies to your research, read the appropriate section before selecting a response.

Materials & experimental systems

n/a	Involved in the study
☒	☐ Antibodies
☒	☐ Eukaryotic cell lines
☒	☐ Palaeontology and archaeology
☒	☐ Animals and other organisms
☒	☐ Clinical data
☒	☐ Dual use research of concern
☒	☐ Plants

Methods

n/a	Involved in the study
☒	☐ ChIP-seq
☒	☐ Flow cytometry
☒	☐ MRI-based neuroimaging

Plants

Seed stocks	NA
Novel plant genotypes	NA
Authentication	NA
